# Genome-wide analysis of NAC transcription factors in grain amaranth reveals structural diversity and regulatory features

**DOI:** 10.1038/s41598-025-23630-7

**Published:** 2025-11-14

**Authors:** Ramesh Eerapagula, Akshay Singh, Avantika Maurya, Rakesh Singh, Nagendra Kumar Singh, Ajay Kumar Mahato

**Affiliations:** 1 Lab of Genome Informatics, BRIC-Centre for DNA Fingerprinting and Diagnostics, Hyderabad, 500039 India; 2 Division of Genomic Resources, ICAR-National Bureau of Plant Genetic Resources, New Delhi, 110012 India; 3 The Genomics Foundation (TGF), Dwarka, New Delhi, 110075 India

**Keywords:** *Amaranthus hypochondriacus*, NAC transcription factors, Abiotic and biotic stress response, Comparative genomics, Gene duplication and evolution, miRNA–target interaction, Genome informatics, Computational biology and bioinformatics, Plant sciences, Plant stress responses

## Abstract

Grain amaranth (*Amaranthus hypochondriacus*) is a climate-resilient pseudo-cereal with high nutritional value and stress tolerance. NAC transcription factors (TFs) are key regulators in plant development and environmental responses. Here, we performed a comprehensive genome-wide analysis of 70 NAC genes (*AhypNACs*), classified into 24 phylogenetic subfamilies. Gene structure analysis showed exon variation (1–7), and chromosomal mapping revealed uneven distribution, with 32 segmental, 5 tandem, and 32 transposed duplications. Promoter analysis identified diverse *cis*-regulatory elements linked to light, hormone signaling, and stress response. miRNA analysis suggested Ahyp-miR164a targets four NAC genes, indicating post-transcriptional control. Comparative genomics showed both conserved and lineage-specific expansions among *Oryza* *sativa*, *Beta vulgaris*, *Arabidopsis thaliana*, and *Chenopodium quinoa.* Transcriptome analysis across seven tissues and five stress conditions identified several condition-specific and multi-stress-responsive NAC genes, with *AhypNAC17, AhypNAC23*, and *AhypNAC24* showing strong induction. RT-qPCR validation under drought stress confirmed early and sustained upregulation of *AhypNAC23* and *AhypNAC24*, and downregulation of *AhypNAC03* and *AhypNAC70*, supporting their functional roles in drought adaptation. This study provides key insights into the structural diversity, evolutionary patterns, and stress-regulatory roles of NAC TFs in *A. hypochondriacus*, laying the foundation for genetic improvement of stress-resilient cultivars.

**Supplementary Information:**

The online version contains supplementary material available at 10.1038/s41598-025-23630-7.

## Introduction

The genus Amaranthus, derived from the Greek word meaning “eternal” or “everlasting,” encompasses species of significant agricultural and nutritional value. Among these, Amaranth, commonly known as Prince’s feather, is recognised as a promising pseudo-cereal and leafy vegetable. Unlike true cereals that store carbohydrates in the endosperm, grain Amaranth accumulates starch in the perisperm, justifying its classification as a pseudo-cereal^1^. Amaranth grains are nutritionally rich, offering high levels of essential micronutrients including iron, calcium, magnesium, β-carotene, folic acid, and vitamin C^2^. The seeds are particularly notable for their elevated protein content (~ 22.5%), substantial dietary fibre (~ 8%), and lysine content (0.73–0.84%), which significantly surpasses that of common cereals such as maize and wheat^3^. In addition to its nutritional benefits, amaranth leaves have demonstrated therapeutic potential by inhibiting the abnormal proliferation of breast, colon, and liver cancer cells, highlighting its value in dietary and medicinal contexts^4^.

Transcription factors are key regulatory proteins that modulate gene expression by binding to specific *cis*-acting elements in the promoter regions of target genes. These proteins orchestrate a wide range of physiological and developmental processes in plants, particularly in response to environmental cues^5^. Based on conserved DNA-binding domains, plant TFs are categorised into 58 families^6^, among them the most prominent families are NAC, MYB, AP2/ERF, HD-Zip, bZIP, WRKY, ARF, and others^7^. The NAC (NAM, ATAF1/2, and CUC2) family, in particular, represents one of the most prominent plant-specific transcription factor family, playing critical roles in diverse biological functions^8^.

The NAC family derives its name from three founding members: NAM (No Apical Meristem), ATAF1/2 (Arabidopsis Transcription Activator Factors), and CUC2 (Cup-shaped Cotyledon 2)^9–11^. Functionally, the NAM domain is vital for apical meristem formation in *Petunia hybrida*^7^, ATAF1/2 act as repressors in defence responses against necrotrophic fungal and bacterial pathogens^12^ and CUC2 is involved in embryogenesis, floral development, and shoot apical meristem formation in *Arabidopsis thaliana*^10^. Beyond these roles, NAC TFs have been implicated in the regulation of secondary cell wall biosynthesis^13^ shoot and root development^14^, organ boundary maintenance^15^, leaf senescence^16^, fruit ripening^17^, and cell cycle control^18,19^.

Genome-wide studies have identified a variable number of NAC genes across plant species, reflecting their evolutionary diversification and functional specialisation. For example, 117 NAC genes have been reported in *Arabidopsis thaliana*, 151 in *Oryza* *sativa* (rice), 152 in *Glycine max* (soybean), 220 in *Gossypium hirsutum* (cotton), 167 in *Musa acuminata* (banana), 82 in *Nelumbo nucifera* (lotus), 79 in *Asparagus officinalis*, 74 in *Fragaria vesca* (strawberry), and 289 in *Saccharum spontaneum* (sugarcane)^20–27^.

The recent availability of a high-quality, chromosome-level genome assembly for *A. hypochondriacus*^28^ has enabled comprehensive genome-wide analyses of key transcription factor families, including NAC. The present study systematically identifies and characterises the complete set of NAC genes in grain Amaranth, providing a detailed investigation of their structural features, phylogenetic relationships, conserved motifs, gene structures, physicochemical properties, subcellular localisation, transmembrane domains, and chromosomal distribution. Additionally, *cis*-regulatory elements were examined to infer potential regulatory mechanisms, and syntenic analyses were performed to explore evolutionary conservation. In silico expression profiling across various tissues and under multiple abiotic and biotic stresses identified several stress-responsive *AhypNAC* genes, of which four drought-inducible candidates were validated via RT-qPCR.

Collectively, this study offers comprehensive insights into the genomic organisation, evolutionary dynamics, and regulatory potential of the NAC gene family in grain amaranth. These findings establish a foundational framework for functional validation of stress-responsive NAC genes and pave the way for future efforts aimed at improving stress resilience and productivity in amaranth and related crops.

## Results

### Identification of NAC genes

A total of 70 NAC genes were identified in the *Amaranthus hypochondriacus* genome through a homology-based search using the PlantTFDB v5.0 database. Their identities were further confirmed by detecting conserved NAC-specific domains via NCBI’s Conserved Domain Database and Pfam. For consistency, the genes were renamed *AhypNAC01* to *AhypNAC70* based on their genomic locations (Table S1). These genes were unevenly distributed across the 16 chromosomes. The highest numbers were found on 1 and 3, each carrying nine genes (12.86%), followed by 4 with eight (11.43%) and 6 with seven (10%). Six genes (8.57%) were located on 2, 5, and 11, while 7 contained four (5.71%). Fewer genes were mapped to 9, 12, and 14 (three each, 4.29%), and to 8 and 10 (two each, 2.86%). The lowest counts were on 15 and 16, with just one gene each (1.43%). Notably, chromosome 13 had no NAC genes. This uneven pattern, especially the absence on chromosome 13, may be linked to genetic, structural, or evolutionary features that shaped the NAC gene arrangement in *A. hypochondriacus*.

### Motif, domain and gene structure analysis

**Fig. 1:**
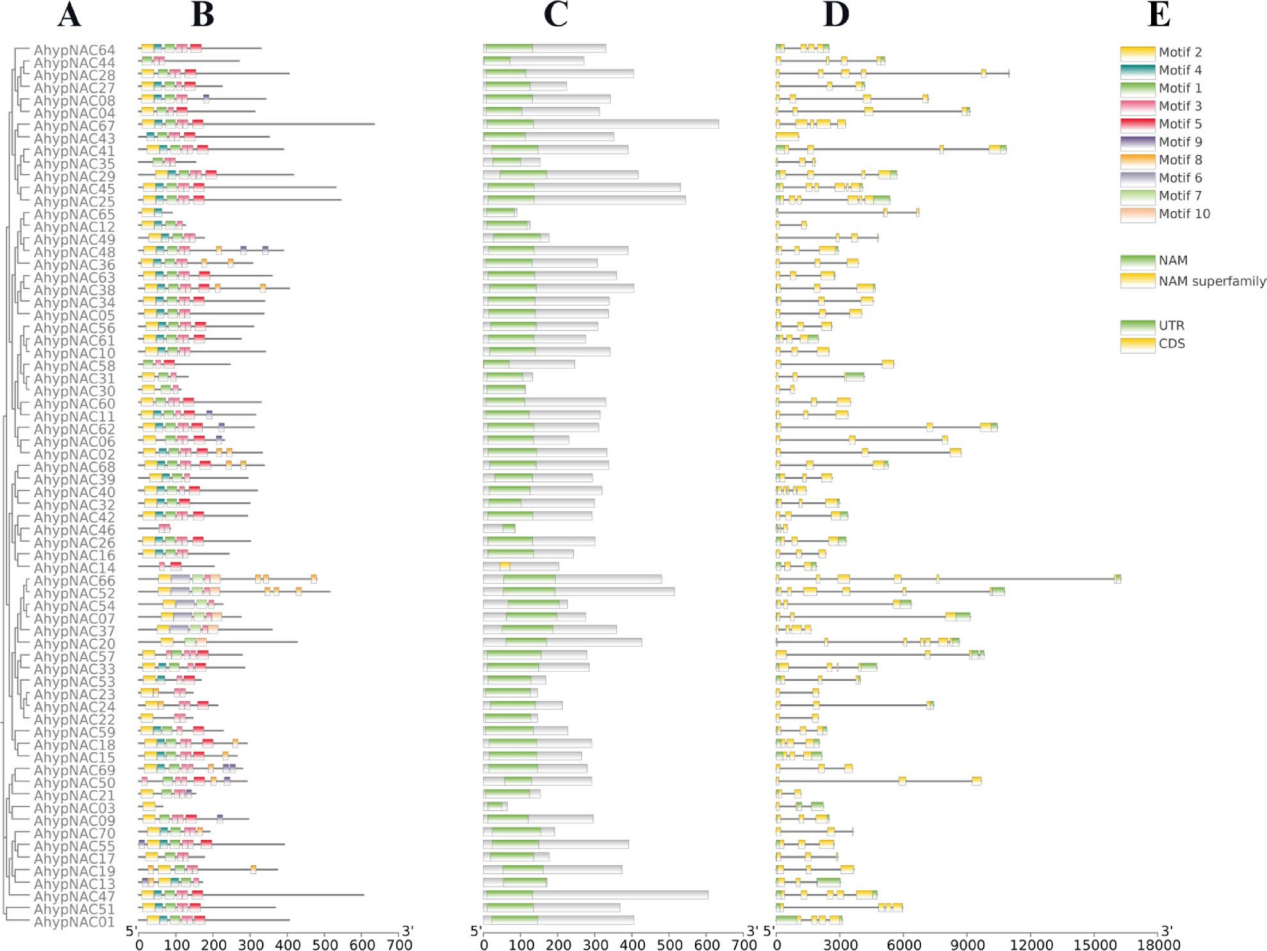
Structural and evolutionary characterization of NAC genes in *Amaranthus hypochondriacus*. (**A**) Phylogenetic tree of 70 AhypNAC proteins constructed using the maximum likelihood method. (**B**) Conserved motif distribution; each coloured box represents a distinct motif, and black lines indicate non-conserved regions. (**C**) Predicted NAC domains highlighted in green. (**D**) Gene structure showing coding sequences (CDS) in yellow and untranslated regions (UTRs) in green. (**E**) Legend for motif and gene structure representations. Nucleotide and amino acid lengths are indicated by scale bars.

All NAC proteins identified in *Amaranthus hypochondriacus* shared a conserved NAC domain at the N-terminal, which is crucial for DNA binding. Most of these proteins contained the five typical subdomains (A–E), with a few exceptions. For instance, AhypNAC65 lacked subdomain E, while AhypNAC14, AhypNAC44, AhypNAC46, and AhypNAC58 missed both A and B (Fig. S1). Motif analysis using MEME revealed 10 conserved regions, ranging from 15 to 50 amino acids in length. Motifs 1 to 5 appeared in most proteins, suggesting core functions, whereas motifs 6, 7, and 10 were found only in specific subgroups. In contrast, motifs 8 and 9 were scattered across different clusters. AhypNAC03 contained just one motif, while a group of 12 genes exhibited six distinct motifs each, indicating structural and possibly functional diversity (Fig. S2). To explore evolutionary relationships, a neighbor-joining (NJ) tree was constructed using the complete NAC gene set, dividing the genes into nine distinct subgroups (Fig. 1A). The clustering was consistent with motif arrangements, particularly those located at the C-terminal (Fig. 1B). Analysis of gene structures revealed variation in exon–intron organization (Fig. 1C). All genes possessed at least two exons and one intron. The majority (around 71%) displayed a typical structure of three to four exons. A simpler structure with two exons was seen in nine genes, while a more complex organization of six exons was found in seven, including *AhypNAC25*, *AhypNAC28*, and *AhypNAC66*. Additionally, single instances of genes with seven and one exon were observed in *AhypNAC20* and *AhypNAC43*, respectively. Genes within the same phylogenetic group generally shared similar structures, highlighting evolutionary conservation (Fig. 1D, E).

### Chromosomal distribution, syntenic relationships, orthologous clustering, and evolutionary dynamics of NAC genes

**Fig. 2:**
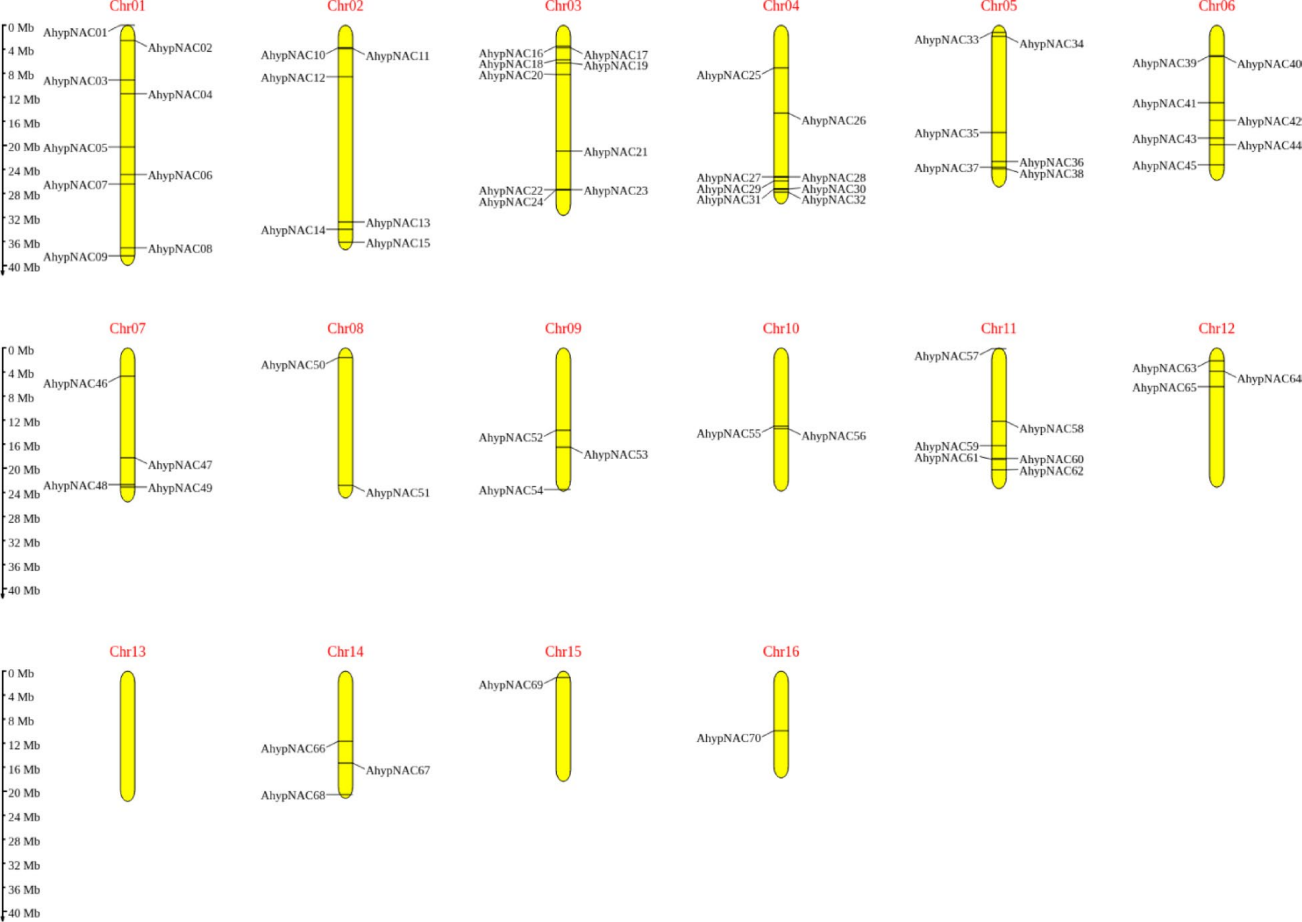
Chromosomal distribution of *AhypNAC* genes across the 16 chromosomes. Chromosome sizes (in megabases, Mb) and gene positions are shown on scale.

The 70 *AhypNAC* genes were unevenly distributed across 16 chromosomes, with the highest numbers on chromosomes 1 and 3 (9 genes each), followed by 4 (8) and 6 (7). Chromosomes 13 lacked NAC genes entirely (Fig. 2). Gene duplication contributed significantly to NAC expansion: 32 genes originated from dispersed duplications, 32 from segmental or whole-genome duplication, and five from tandem duplications. One gene (*AhypNAC28*) likely arose via proximal duplication. Segmental duplications (17 gene pairs) had Ka/Ks ratios between 0.12 and 0.43, suggesting purifying selection (Fig. S2, Table S2-S3).

**Fig. 3:**
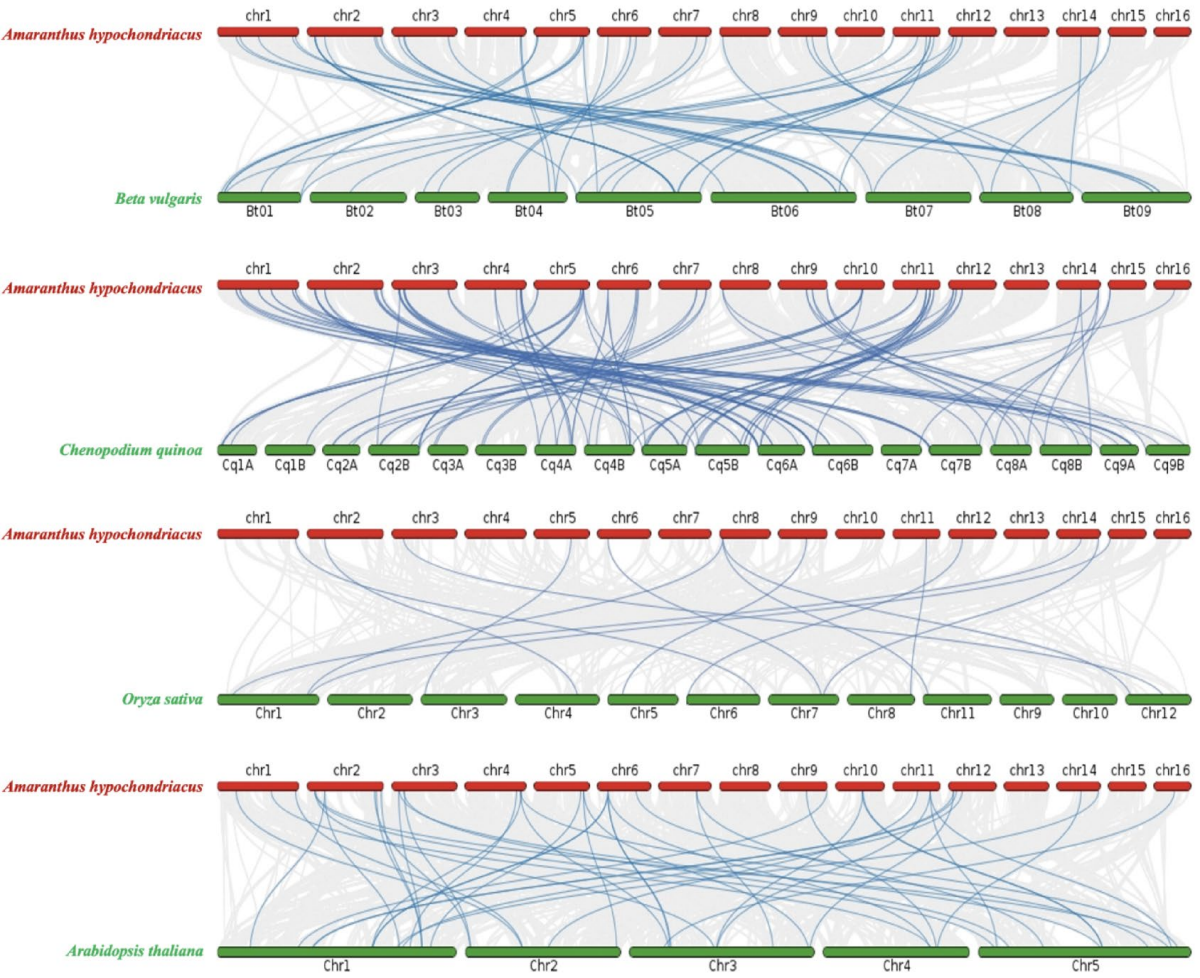
Syntenic relationship of *AhypNAC* genes with *B. vulgaris*, *C. quinoa*, *O. sativa*, and *A. thaliana*. Grey lines indicate syntenic blocks across genomes, while blue lines represent syntenic NAC gene pairs.

Synteny analysis revealed conserved NAC loci between *A. hypochondriacus* and four species. With *C. quinoa,* 104 syntenic NACs were found, predominantly on chromosomes 1 and 2. Comparisons with *A. thaliana* identified 46 syntenic genes, with strongest matches from chromosome 2. Moderate conservation was observed with *O. sativa* (14 genes), while *B. vulgaris* shared 21 genes, mainly from chromosome 2. Chromosomes 13 and 16 showed no conservation across species, reflecting divergence (Fig. 3).

**Fig. 4:**
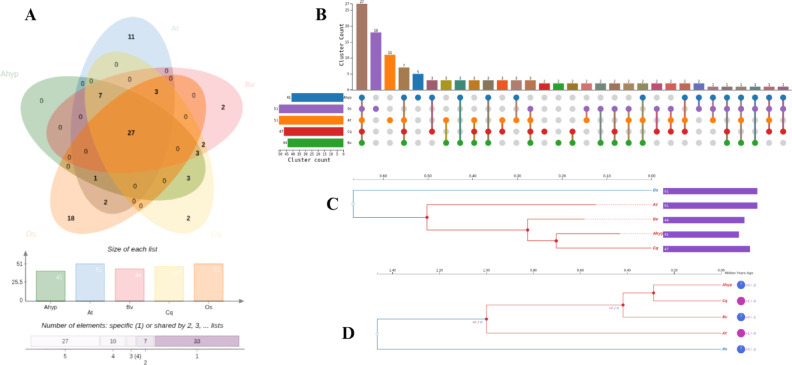
Comparative genomic and evolutionary analysis of NAC transcription factors across five plant species: *Amaranthus hypochondriacus* (Ahyp), *Arabidopsis thaliana* (At), *Oryza* *sativa* (Os), *Beta vulgaris* (Bv), and *Chenopodium quinoa* (Cq). (**A**) Venn diagram showing shared and unique orthogroups among the five species, highlighting conserved and lineage-specific NAC gene sets. (**B**) Upset plot illustrating the intersections of orthologous groups across species, providing insight into cluster-level conservation and divergence. (**C**) Unrooted phylogenetic tree constructed using the maximum likelihood method (JTT + CAT model), depicting evolutionary relationships among representative NAC orthogroups. **(D**) Ultrametric phylogenetic tree generated by CAFE, showing gene family expansion (magenta) and contraction (blue) events across lineages, with numeric values indicating the number of affected orthogroups.

Orthologous clustering using OrthoVenn3 across five species (*A. hypochondriacus* (Ahyp), *A. thaliana* (At), *O. sativa* (Os), *B. vulgaris* (Bv), *C. quinoa* (Cq)) identified 81 orthogroups. *A. hypochondriacus* shared 27 core NAC genes with all species, suggesting conserved ancestral functions. Notably, three genes were uniquely shared with *C. quinoa*, indicating a closer evolutionary relationship. No NAC genes were exclusive to Ahyp alone. *O. sativa* displayed the highest number of species-specific orthologs (18), consistent with monocot-dicot divergence. A total of 76 singleton genes were identified, including 18 from Ahyp, suggesting possible lineage-specific innovations (Fig. 4A, B).

Phylogenetic analysis using the maximum likelihood method (JTT + CAT model) revealed that *A. hypochondriacus* is most closely related to *C. quinoa* (bootstrap support = 0.890), with both species clustering within the Caryophyllales clade. *B. vulgaris* was more distantly related, while *A. thaliana* and *O. sativa* were placed in more basal positions, with the latter being the most divergent, reflecting its monocot origin (Fig. 4C).

Gene family evolution analysis using CAFE (Computational Analysis of gene Family Evolution) indicated dynamic changes. *C. quinoa* showed the highest expansion (+ 2 orthogroups), *A. thaliana* showed moderate expansion (+ 1), while *A. hypochondriacus* and *O. sativa* each exhibited contraction in two orthogroups. These patterns highlight lineage-specific gene gains or losses, likely driven by adaptive evolution or genome restructuring (Fig. 4D).

### Phylogenetic analysis

**Fig. 5:**
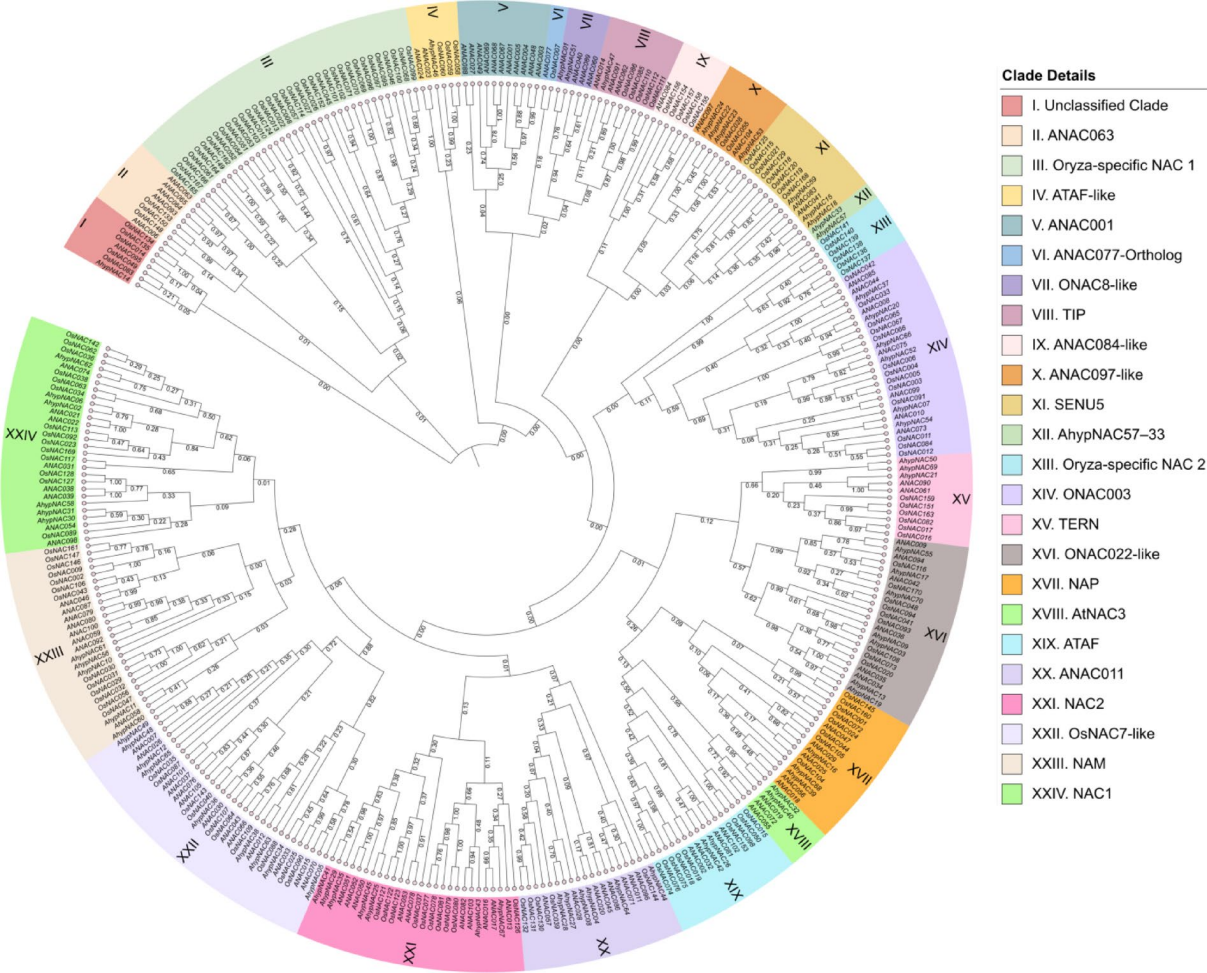
Phylogenetic tree of NAC proteins from *A. hypochondriacus, A. thaliana*, and *O. sativa* constructed using the maximum likelihood method (JTT model, 1,000 bootstraps). The 272 proteins clustered into 24 clades, including known and novel subfamilies. Coloured branches represent subgroups; internal nodes show bootstrap values, reflecting evolutionary relationships and lineage-specific diversification.

A phylogenetic tree was constructed using 272 NAC protein sequences from *Amaranthus hypochondriacus* (AhypNAC—70), *Arabidopsis thaliana* (ANAC—105), and *Oryza* *sativa* (OsNAC—97), employing the maximum likelihood method (Jones-Taylor-Thornton (JTT) model) with 1000 bootstrap replicates. The NAC proteins clustered into 24 clades, highlighting both conserved and species-specific groups. Clade I comprised seven divergent NACs, including AhypNAC14, ANAC095, and OsNAC134, which lacked strong subfamily assignments and may represent fast-evolving genes. Clades II (ANAC063), III (Oryza-specific NAC 1), and IV (ATAF-like) included conserved regulators of stress responses, with Clade IV featuring ANAC023/024 and their orthologs. Clade V (ANAC001) and VI (ANAC077-Ortholog) consisted of evolutionarily preserved pairs. Clade VII (ONAC8-like) and VIII (TIP) were linked to development and membrane trafficking. Clade IX (ANAC084-like) and X (ANAC097-like) retained conserved Arabidopsis sequences, while Clade XI (SENU5), anchored by ANAC083, included 13 genes like OsNAC118–120 and AhypNAC59, associated with oxidative stress regulation (Fig. 5).

Clade XII, with AhypNAC33 and AhypNAC57, appeared unique to *A. hypochondriacus*. Clade XIII and Clade XIV contained canonical regulators like ANAC008. Clade XV (TERN) included TM-NACs with membrane localization. Clade XVI (ONAC022-like) was broadly conserved, possibly regulating development and senescence. Clade XVII (NAP), containing ANAC029 and its amaranth homologs, was associated with leaf aging and hormonal signaling. Clade XVIII (AtNAC3), centered on ANAC019, grouped AhypNAC40 and AhypNAC32 likely functioning in abiotic stress and senescence. Clade XIX (ATAF) included 15 genes from all three species, including stress-linked repressors like ANAC002 and OsNAC075. Clade XX (ANAC011), with 19 members, was broadly conserved, possibly involved in developmental regulation. Clade XXI (NAC2), XXII (OsNAC7-like), and XXIII (NAM) contained developmental regulators involved in root and shoot development. Clade XXIV (NAC1) was the largest, with 28 members, including NACs involved in auxin signaling and organogenesis, supporting its functional importance across species.

### Physiochemical properties, subcellular localisation and transmembrane helices

To assess functional diversity within the AhypNAC protein family, 70 NAC proteins were analyzed using the Multiple Protein Profiler and TMHMM tools. The assessment included protein length, molecular weight (MW), isoelectric point (pI), hydropathicity (GRAVY), aliphatic and instability indices, aromaticity, net charge at pH 7.0, subcellular localization, and potential transmembrane domains. Protein lengths ranged from 65 (AhypNAC03) to 634 amino acids (AhypNAC67), with corresponding MWs of 7.75 to 70.27 kDa. The predicted pI values varied between 4.51 and 9.93, grouping 34 proteins as acidic (pI < 6.5), 11 as near-neutral (6.5–7.5), and 25 as basic (pI > 7.5), indicating charge diversity under physiological conditions. All proteins exhibited negative GRAVY values (− 1.183 to − 0.16), confirming their hydrophilic nature. The aliphatic index ranged from 42.43 to 88.49, while instability indices spanned 23.90 to 77.05, suggesting that 41 proteins are potentially unstable under in vitro conditions. Aromaticity values remained low (0.05–0.15), and net charge fluctuated widely (− 35.51 to + 11.67), implying differences in electrostatic properties and interaction capabilities. Subcellular localization prediction placed 64 proteins in the nucleus, consistent with their expected roles as transcription factors. Six proteins were predicted to localize to organelles, including the chloroplast *(*AhypNAC35, 46, 54) and endomembrane system (AhypNAC25, 45, 67) (Table 1). In addition, TMHMM analysis identified transmembrane helices in six proteins, suggesting possible involvement in membrane-associated functions (Table S4).

**Table 1:** Physiochemical properties and subcellular localisation predictions of *A. hypochondriacus* NAC proteins.

Protein ID	Length(aa)	GRAVY	Aliphatic index	Instability index	Stability	MW (kDa)	Aromaticity	pI	Charge at pH 7	Localization
*AhypNAC01*	406	− 0.55	77.07	52.86	Unstable	45.36	0.07	8.58	5.34	Nucleus
*AhypNAC02*	333	− 0.89	58.8	42.57	Unstable	38.17	0.1	6.38	− 4.7	Nucleus
*AhypNAC03*	65	− 0.64	64.46	77.05	Unstable	7.75	0.12	6.82	− 0.23	Nucleus
*AhypNAC04*	313	− 0.77	58.59	35.31	Stable	35.87	0.1	5.48	− 7.89	Nucleus
*AhypNAC05*	337	− 0.97	52.73	33.07	Stable	38.64	0.09	7.14	0.57	Nucleus
*AhypNAC06*	231	− 0.9	56.49	42.64	Unstable	26.59	0.11	8.76	3.83	Nucleus
*AhypNAC07*	276	− 0.98	57.5	35.25	Stable	31.25	0.08	9.05	8.42	Nucleus
*AhypNAC08*	342	− 0.78	63.33	37.9	Stable	39.12	0.1	5.06	− 14.7	Nucleus
*AhypNAC09*	296	− 0.71	71.11	46.11	Unstable	33.75	0.09	9.1	5.21	Nucleus
*AhypNAC10*	341	− 0.73	60.03	43.47	Unstable	38.54	0.11	6.01	− 4.72	Nucleus
*AhypNAC11*	315	− 0.66	66.57	39.47	Stable	35.65	0.11	5.97	− 1.43	Nucleus
*AhypNAC12*	126	− 0.62	71.98	38.51	Stable	14.79	0.15	8.91	2.85	Nucleus
*AhypNAC13*	172	− 0.99	62.91	39.29	Stable	20.12	0.1	5.95	− 2.22	Nucleus
*AhypNAC14*	203	− 0.64	80.15	43.4	Unstable	23.21	0.08	8.8	3.02	Nucleus
*AhypNAC15*	265	− 0.8	60.38	41.32	Unstable	29.99	0.09	7.19	0.54	Nucleus
*AhypNAC16*	243	− 0.65	68.64	32.79	Stable	27.7	0.12	7.72	1.02	Nucleus
*AhypNAC17*	177	− 0.83	65.54	39.49	Stable	20.84	0.12	9.4	8.92	Nucleus
*AhypNAC18*	292	− 0.81	61.1	51.31	Unstable	33.63	0.11	7.66	1.29	Nucleus
*AhypNAC19*	374	− 0.76	63.07	45.89	Unstable	42.06	0.08	6.49	− 3	Nucleus
*AhypNAC20*	427	− 0.75	68.71	48.97	Unstable	47.97	0.07	5.09	− 17.63	Nucleus
*AhypNAC21*	153	− 1.06	45.23	63.21	Unstable	17.89	0.14	9.4	4.69	Nucleus
*AhypNAC22*	146	− 0.22	80.75	53.74	Unstable	16.87	0.12	9.12	4.01	Nucleus
*AhypNAC23*	146	− 0.71	55.41	63.01	Unstable	17.13	0.14	8.67	3.26	Nucleus
*AhypNAC24*	213	− 0.68	62.21	59.74	Unstable	25.01	0.12	5.81	− 7.48	Nucleus
*AhypNAC25*	545	− 0.44	70.95	40.13	Unstable	61.01	0.11	4.59	− 35.51	Endomembrane system
*AhypNAC26*	301	− 0.66	54.78	45.66	Unstable	34	0.11	7.04	0.09	Nucleus
*AhypNAC27*	225	− 0.69	62	39.5	Stable	25.7	0.11	5.76	− 4.09	Nucleus
*AhypNAC28*	405	− 0.82	57.78	46.34	Unstable	46.6	0.1	5.94	− 9.05	Nucleus
*AhypNAC29*	417	− 0.6	73.65	58.64	Unstable	46.76	0.07	5.53	− 7.99	Nucleus
*AhypNAC30*	114	− 0.83	68.42	34.89	Stable	13.29	0.11	9.88	7.69	Nucleus
*AhypNAC31*	133	− 0.94	52.78	29.94	Stable	15.27	0.13	9.89	11.67	Nucleus
*AhypNAC32*	299	− 0.5	69.46	31.09	Stable	33.3	0.11	6.9	− 0.17	Nucleus
*AhypNAC33*	286	− 0.48	68.53	51.35	Unstable	32.3	0.11	7.56	1.06	Nucleus
*AhypNAC34*	339	− 0.84	62.39	36.83	Stable	38.8	0.11	6.87	− 0.45	Nucleus
*AhypNAC35*	153	− 0.33	77.12	23.9	Stable	17.78	0.12	9.36	6.65	Chloroplast
*AhypNAC36*	307	− 0.97	60.65	49.18	Unstable	36.36	0.12	6.5	− 4.23	Nucleus
*AhypNAC37*	359	− 0.68	71.95	37.66	Stable	39.86	0.07	5.21	− 16.16	Nucleus
*AhypNAC38*	406	− 0.76	68.18	45.19	Unstable	46.15	0.08	5.94	− 10.66	Nucleus
*AhypNAC39*	294	− 0.72	65.65	50.04	Unstable	32.52	0.07	5.77	− 6.6	Nucleus
*AhypNAC40*	320	− 0.68	61.53	38.72	Stable	35.89	0.11	6.61	− 0.96	Nucleus
*AhypNAC41*	390	− 0.61	70.26	45.05	Unstable	43.55	0.08	5.51	− 8	Nucleus
*AhypNAC42*	293	− 0.63	66.25	44.97	Unstable	33.47	0.12	7.15	0.26	Nucleus
*AhypNAC43*	351	− 0.41	72.79	29.16	Stable	38.52	0.09	4.76	− 16.98	Nucleus
*AhypNAC44*	271	− 0.72	53.91	40.43	Unstable	30.85	0.11	5.57	− 7.73	Nucleus
*AhypNAC45*	531	− 0.44	78.17	35.71	Stable	59.83	0.09	4.81	− 27.53	Endomembrane system
*AhypNAC46*	86	− 0.16	88.49	26.52	Stable	9.76	0.12	9.93	6.75	Chloroplast
*AhypNAC47*	606	− 0.58	72.36	47.68	Unstable	67.81	0.08	5.04	− 22.54	Nucleus
*AhypNAC48*	390	− 1.05	60.97	47.24	Unstable	45.39	0.09	6.07	− 8.66	Nucleus
*AhypNAC49*	177	− 0.99	60.56	39.86	Stable	20.78	0.13	6.01	− 2.8	Nucleus
*AhypNAC50*	292	− 1.18	42.43	50.32	Unstable	33.19	0.1	6.39	− 1.9	Nucleus
*AhypNAC51*	368	− 0.54	72.28	45.18	Unstable	41.79	0.09	8.76	6.27	Nucleus
*AhypNAC52*	515	− 0.93	56.45	49.94	Unstable	57.72	0.06	6.52	− 7.22	Nucleus
*AhypNAC53*	168	− 0.81	68.39	45.69	Unstable	19.62	0.12	4.51	− 16.06	Nucleus
*AhypNAC54*	227	− 0.6	74.23	37.9	Stable	25.52	0.07	9.1	7.6	Chloroplast
*AhypNAC55*	392	− 0.53	69.67	52.96	Unstable	44.14	0.1	7.53	0.97	Nucleus
*AhypNAC56*	309	− 0.69	60.87	34.2	Stable	35.22	0.1	5.22	− 9.96	Nucleus
*AhypNAC57*	279	− 0.38	78.17	50.28	Unstable	31.63	0.11	7.59	1.32	Nucleus
*AhypNAC58*	246	− 0.67	62.11	25.29	Stable	27.72	0.1	9.18	5.11	Nucleus
*AhypNAC59*	228	− 0.76	56.4	62.41	Unstable	26.81	0.14	5.24	− 10.71	Nucleus
*AhypNAC60*	330	− 0.74	64.39	38.36	Stable	37.44	0.11	6.23	− 2.06	Nucleus
*AhypNAC61*	276	− 0.72	57.21	34.15	Stable	31.52	0.13	8.85	4.92	Nucleus
*AhypNAC62*	311	− 0.75	62.38	40.66	Unstable	35.64	0.1	6.11	− 5.39	Nucleus
*AhypNAC63*	359	− 0.87	64.62	46.04	Unstable	41.61	0.09	5.99	− 8.05	Nucleus
*AhypNAC64*	330	− 0.61	59.67	43.11	Unstable	37.39	0.13	5.51	− 6.08	Nucleus
*AhypNAC65*	91	− 0.94	63.19	36.74	Stable	10.98	0.15	5.88	− 2.22	Nucleus
*AhypNAC66*	480	− 0.92	62.17	55.44	Unstable	54.22	0.05	6.29	− 10.2	Nucleus
*AhypNAC67*	634	− 0.48	71.66	44.72	Unstable	70.27	0.1	4.77	− 29.61	Endomembrane system
*AhypNAC68*	338	− 0.8	63.43	39.44	Stable	38.38	0.09	7.35	1.16	Nucleus
*AhypNAC69*	280	− 1.12	59.21	60.14	Unstable	32.33	0.1	7.75	1.21	Nucleus
*AhypNAC70*	192	− 0.88	67.5	26.85	Stable	22.28	0.1	9.24	7.24	Nucleus

### *Cis*-Regulatory elements and miRNA-mediated regulation of NAC genes

**Fig. 6:**
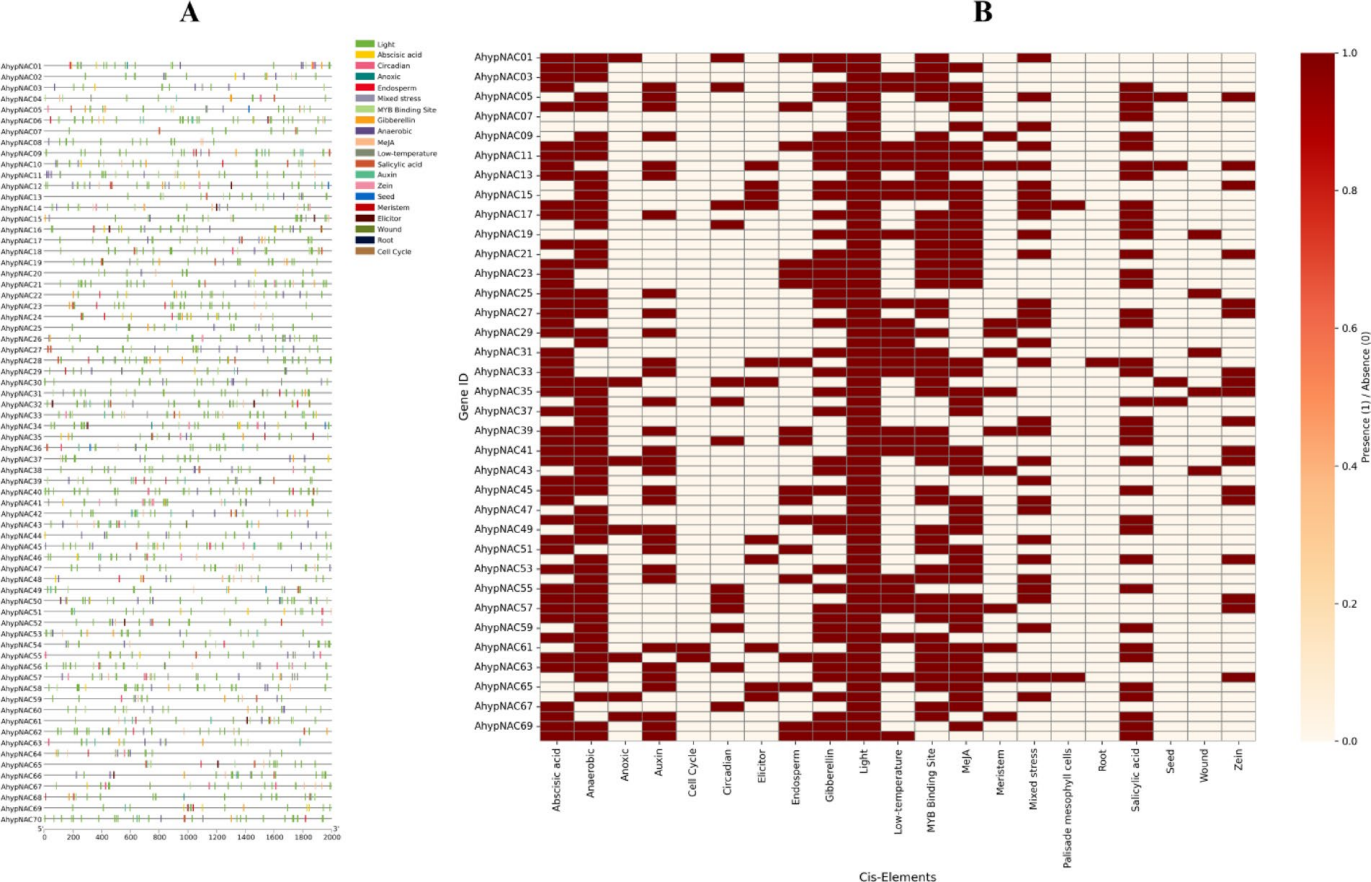
*Cis*-acting elements in AhypNAC gene promoters of *A. hypochondriacus*. (**A**) Functional categorization of elements within 2000 bp upstream regions (light, hormone, stress, development). (**B**) Heatmap highlighting element presence (red) and regulatory diversity across genes.

To explore transcriptional regulation of the 70 *AhypNAC* genes, we analyzed their 2000 bp upstream promoter regions and identified over 1700 *cis*-acting elements classified into 20 functional categories. Light-responsive elements were most prevalent (902 instances; 51.84%), including G-box, GATA-motif, Sp1, and TCT-motif, suggesting light-mediated control of NAC expression in *A. hypochondriacus*. Hormone-responsive motifs were also abundant: jasmonate-related elements (TGACG and CGTCA) occurred 152 times (8.74%), abscisic acid-related ABRE motifs appeared 125 times (7.18%), while motifs for gibberellin (3.22%), auxin (2.6%), and salicylic acid (2.59%) were also detected. Stress-related elements, such as anaerobic response elements (AREs, 100 instances), TC-rich repeats, and LTR motifs, indicate potential roles in hypoxia and cold stress adaptation. Developmental regulatory motifs like CAT-box (meristem activity), GCN4 (endosperm-specific), and MSA-like elements (cell cycle) were also present. Additionally, 86 MYB-binding motifs (4.94%) were identified, indicating possible co-regulation by MYB and NAC transcription factors (Fig. 6A, B).

To investigate post-transcriptional control, we predicted miRNA targets and identified Ahyp-miR164a as a conserved regulator of four NAC genes—*AhypNAC02*, *AhypNAC10*, *AhypNAC56*, and *AhypNAC61*. All interactions were cleavage-based, with binding sites located within the coding regions (569–703 bp). Expectation values ranged from 0.5 to 3.0 and UPE scores from 7.49 to 17.82, indicating stable interactions (Table S5). These results suggest miR164a-mediated fine-tuning of NAC gene expression in stress or development-related pathways.

### Comprehensive expression profiling and qPCR validation of *AhypNAC* genes

**Fig. 7:**
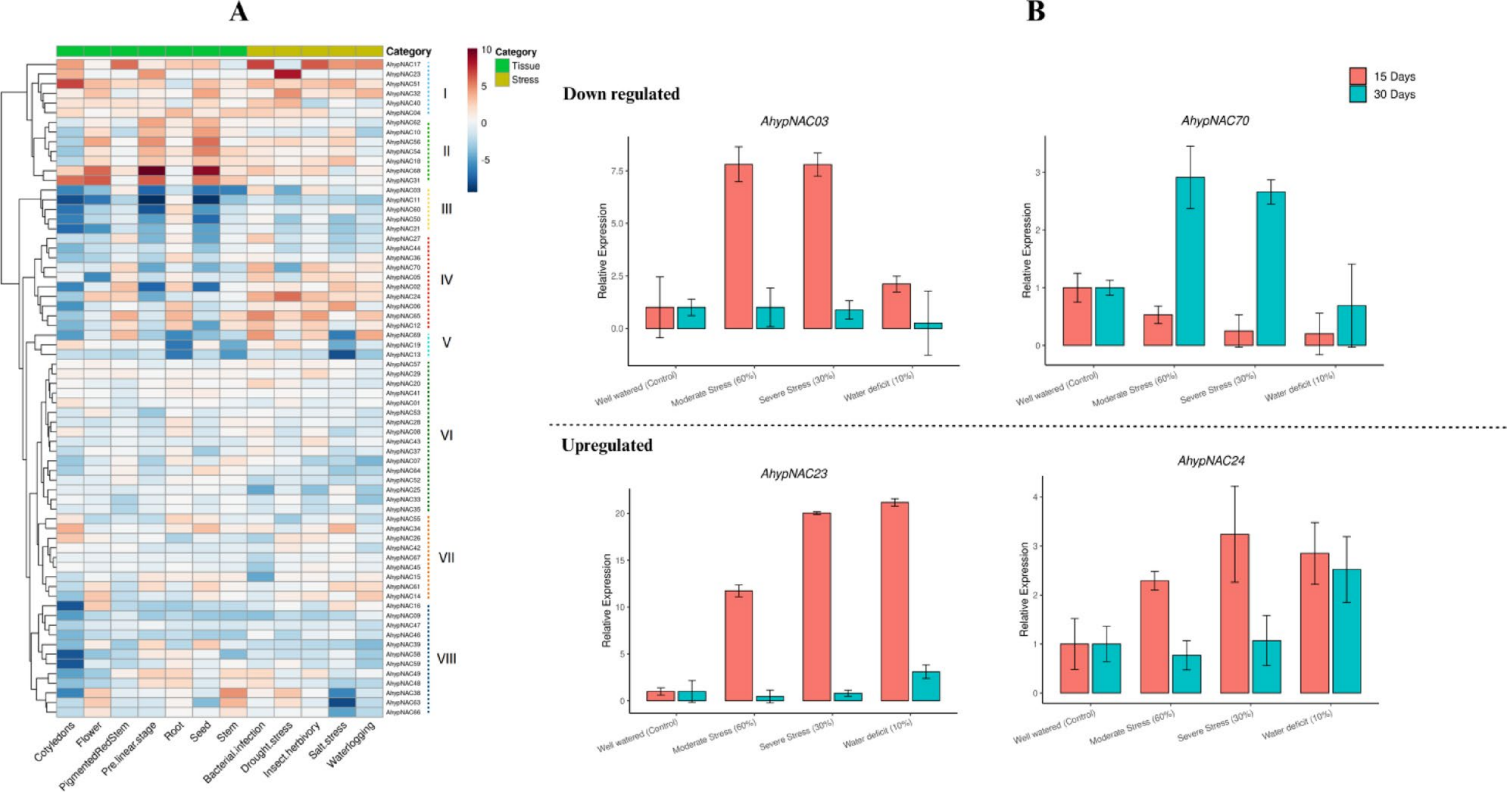
Heatmap showing log₂ fold-change expression of 70 *AhypNAC* genes across eight tissues and five stress conditions in *Amaranthus hypochondriacus*. (**A**) Genes are grouped into eight clusters (I–VIII) based on expression similarity. Red and blue indicate high and low expression, respectively. Top colour bar marks sample type: green (tissue) and yellow (stress). (**B**) qRT-PCR validation of *AhypNAC03*, *AhypNAC70*, *AhypNAC23* and *AhypNAC24* under different water stress.

Comprehensive transcriptome analysis of 70 *AhypNAC* genes across seven tissues and five stress conditions revealed distinct expression signatures, classified into eight co-expression clusters (I–VIII), suggesting functional divergence in development and stress response in *A. hypochondriacus*. In cotyledons, *AhypNAC51*, *17* (Cluster I), and *31* (Cluster II) exhibited strong expression, while *AhypNAC03* (III), *21* (III), *02* (IV), *16* (VIII), *58* (VIII), *59* (VIII), and *60* (III) showed low expression levels. Flowers showed high expression of *AhypNAC68* (II) and *31* (II), with *AhypNAC11* (III), *21* (III), and *05* (IV) expressed at lower levels. In pigmented red stem, *AhypNAC17* (I) was highly expressed, while *AhypNAC11* (III) showed moderately reduced expression. During the pre-linear stage, *AhypNAC86* (not listed, possibly *AhypNAC68*) and *31* (II) were prominent, whereas *AhypNAC03* (III), *11* (III), *60* (III), and *02* (IV) were less expressed. In the root tissue, genes in Cluster IV, such as *AhypNAC06*, *04*, and *65*, showed high expression, whereas *AhypNAC69* (V), *19* (V), and *13* (V) were minimally expressed. In seeds, *AhypNAC68* (II) and *31* (II) were again strongly expressed, while *AhypNAC11* (III), *50* (III), and *02* (IV) showed reduced levels. Stem-specific expression was observed for *AhypNAC38* (VIII) and *63* (VIII), while *AhypNAC03* (III), *13* (V), and *19* (V) remained low (Fig. 7A, Table S6).

Under drought stress, Cluster I genes (*AhypNAC23*, *24*, *32*) were highly expressed, while *AhypNAC03* (III) and *70* (IV) were downregulated. In salt stress, *AhypNAC06* (IV) and *51* (I) were induced, while genes such as *AhypNAC69* (V), *19* (V), *13* (V), *38* (VIII), *63* (VIII), and *66* (VIII) were suppressed. Waterlogging strongly upregulated *AhypNAC17* (I), *32* (I), and *69* (V), with downregulation of *AhypNAC11* (III), *39* (VIII), *58* (VIII), and *59* (VIII). In bacterial infection, genes from Clusters I and IV such as *AhypNAC17*, *65*, and *69* (V) were upregulated, while *AhypNAC15* (VII) and *25* (VI) were suppressed. Insect herbivory induced *AhypNAC17* (I) and *65* (IV), while *AhypNAC25* (VI) was downregulated. Hierarchical clustering revealed functional modules: Clusters I–II encompassed highly expressed genes across both stress and developmental contexts, while Clusters V–VIII included genes with more specialized or suppressed expression. These transcriptional patterns underscore the regulatory versatility of *AhypNAC* genes and identify core candidates for further functional characterization under environmental and developmental contexts.

To validate in silico predictions, four *AhypNAC* genes were analyzed by RT-qPCR in the drought-tolerant genotype *A. hypochondriacus* “Annapurna” under severe water deficit (10% irrigation). *AhypNAC23* showed strong early induction (~ 21-fold at 15 days) that declined to ~ 3-fold by 30 days, suggesting a transient drought response. *AhypNAC24* remained stably upregulated (~ 3-fold), indicating sustained activation. Conversely, *AhypNAC03* declined from 2.1-fold to 0.25-fold, and *AhypNAC70* remained suppressed, implying negative regulation under prolonged drought. These results support the roles of *AhypNAC23* and *AhypNAC24* as potential positive regulators of drought tolerance (Fig. 7B).

## Discussion

The NAC TF family constitutes one of the largest and most functionally diverse gene families in plants, playing pivotal roles in development, stress response, and senescence regulation^14^. In this study, we identified and characterized 70 NAC genes (*AhypNACs*) in *Amaranthus hypochondriacus*, a climate-resilient pseudo-cereal, and performed a genome-wide analysis of their structural, evolutionary, and expression features. The number of NAC genes in *A. hypochondriacus* is comparable to that in *Arabidopsis thaliana* (150), *Oryza* *sativa* (75), *Capsicum annuum* (104), and *Solanum lycopersicum* (88), *Asparagus officinalis* (85), *Fagopyrum tataricum* (80) suggesting a conserved repertoire across angiosperms^20,25,29–31^. Uneven chromosomal distribution—with no NAC genes found on chromosome 13 alongside identification of 32 segmentally duplicated and five tandemly duplicated genes indicates that gene duplication events have been key drivers of NAC expansion, as also observed in *Glycine max*^32^, *Beta* *vulgaris*^33^, and *Chenopodium* *quinoa*^34^. Gene structure analysis showed variation in exon number (1–7), with most genes containing 3–4 exons, consistent with patterns in *Brassica* *napus*^35^ and *Triticum aestivum*^36^. Promoter analysis revealed a predominance of light-responsive *cis*-elements (~ 52%), along with abundant hormone- and stress-related motifs, particularly ABA and jasmonate elements. This suggests potential cross-talk between light signaling and abiotic stress responses, a pattern also reported in *Arabidopsis*^20^ and *Capsicum annuum*^29^. Phylogenetic and orthologous clustering classified *AhypNACs* into 24 clades, with the OsNAC7-like group showing lineage-specific expansion, indicative of Amaranthaceae specific adaptations. Synteny analysis revealed a high degree of conservation with *C. quinoa*, supporting close evolutionary ties within the Amaranthaceae–Chenopodiaceae clade. The presence of *A. hypochondriacus*-specific orthogroups further implies species-specific functional diversification, while the relatively fewer singletons in *A. hypochondriacus* versus *O. sativa* suggests reduced NAC gene turnover in dicots. Subcellular localization predicted that most AhypNAC proteins are nuclear, consistent with their role as TFs. However, six genes encoding transmembrane domains likely represent membrane-bound NACs (NTLs), similar to those involved in ER stress signaling in *A. thaliana*^20^.

Transcriptome profiling across eight tissues and five stress conditions revealed diverse spatial and temporal expression signatures, grouped into eight co-expression clusters. Tissue-specific expression of genes like *AhypNAC68* and *AhypNAC31* (flower/seed) and *AhypNAC06* and *AhypNAC65* (root) points to specialized developmental functions. Stress-induced expression analysis revealed that *AhypNAC23, AhypNAC24*, and *AhypNAC32* were strongly upregulated under drought, while *AhypNAC17* and *AhypNAC69* were induced across multiple stress conditions (salt, bacterial, insect, and waterlogging), suggesting their role as central regulators in the stress response network. Similar multi-stress NAC regulators have been reported in *Musa acuminata*^23^ and *Zea mays*^37^. RT-qPCR validation under drought stress further confirmed the transcriptomic findings. *AhypNAC23* exhibited strong early induction (~ 21-fold at 15 days), while *AhypNAC24* showed sustained upregulation, aligning with drought-responsive patterns reported for *AmNAC24* in *Ammopiptanthus mongolicus*^38^, which is implicated in ROS scavenging through upregulation of antioxidant enzymes^39^. The downregulation of *AhypNAC03* and *AhypNAC70* under prolonged drought mirrors findings in peanut, where *AhNAC3* homologs regulate downstream drought-related genes^40^. Together, these results highlight the functional divergence and regulatory complexity of NAC genes in A. hypochondriacus, offering valuable candidates for stress-resilient crop improvement. Notably, the identified drought- and multi-stress-responsive NACs particularly *AhypNAC17*, *AhypNAC23*, and *AhypNAC24* serve as promising targets for future CRISPR-Cas9-based gene editing and transgenic overexpression assays. These approaches will be essential for functionally validating candidate NACs and translating these findings into practical applications for molecular breeding and the development of climate-resilient amaranth cultivars.

## Conclusion

In this study, 70 NAC transcription factor genes were identified in *Amaranthus hypochondriacus* and comprehensively analysed for their chromosomal distribution, gene structure, duplication patterns, phylogenetic classification, and regulatory features. Expression profiling across various tissues and five abiotic and biotic stress conditions, supported by RT-qPCR validation, revealed key drought-responsive genes including *AhypNAC23*, *AhypNAC24*, *AhypNAC03*, and *AhypNAC70*. Predictions of transmembrane domains and miRNA interactions, particularly with Ahyp-miR164a, indicate multilayered regulatory control. These findings suggest that several *AhypNAC* genes play important roles in drought and multi-stress tolerance. This work offers a valuable genomic resource for future functional studies and provides a strong foundation for CRISPR-Cas9-based gene editing or overexpression assays aimed at developing climate-resilient amaranth cultivars through molecular breeding.

## Materials and methods

### Identification of NAC genes

The genome, protein, coding sequences, and annotation files of *A. hypochondriacus* were downloaded from Phytozome (https://phytozome-next.jgi.doe.gov/) submitted under Phytozome genome ID: 459^41^. To identify the NAC genes in the amaranth genome, the downloaded protein sequences were subjected to a sequence similarity search against the PlantTFDB v5.0 (https://planttfdb.gao-lab.org/download.php)^6^. The identified NAC protein sequences were further validated in silico to ensure the presence of the NAC domain using the NCBI Conserved Domain Database (CDD)^42^, InterProScan^43^ and the Pfam protein domain database^44^.

### Sequence alignment, phylogenetic relationships, conserved motifs and gene structures

NAC protein sequences of *A. hypochondriacus*, *A. thaliana* (https://www.arabidopsis.org/) and *O. sativa*(https://phytozome-next.jgi.doe.gov/info/Osativa_v7_0) were aligned using MUSCLE (MEGA v11.0.13)^45^. A phylogenetic tree was constructed using the maximum likelihood method (JTT model, 1000 bootstraps) and visualized with iTOL^46^. Conserved motifs were identified using MEME (maximum motifs = 10), and gene structures were extracted from GFF files and visualized using TBtools^47^.

### Chromosomal mapping, synteny, duplication, and orthologous cluster analysis

NAC protein sequences of *A. hypochondriacus*, *A. thaliana* (https://www.arabidopsis.org/) and *O. sativa*(https://phytozome-next.jgi.doe.gov/info/Osativa_v7_0) were aligned using MUSCLE (MEGA v11.0.13)^45^. A phylogenetic tree was constructed using the maximum likelihood method (JTT model, 1000 bootstraps) and visualized with iTOL^46^. Conserved motifs were identified using MEME (maximum motifs = 10), and gene structures were extracted from GFF files and visualized using TBtools^47^.

### Physiochemical properties, subcellular localisation, and transmembrane helices prediction

The physiochemical properties of the identified NAC proteins were identified using Multiple Protein Profiler v1.0.^48^, subcellular localisation was predicted with BUSCA^49^ while transmembrane helices were identified using TMHMM v2.0^50^ highlighting potential membrane-associated NACs.

### Cis-Regulatory elements and miRNA analysis

The Promoter regions (2000 bp upstream) of *AhypNAC* genes were analyzed using PlantCARE^51^ to identify *cis*-acting regulatory elements, with visualization via TBtools. To explore post-transcriptional regulation, miRNA–NAC interactions were predicted using psRNATarget^52^, employing *A. hypochondriacus* specific mature miRNAs from Martínez Núñez et al.^53^, revealing potential miRNA binding sites under default parameters, including an expectation score of ≤ 3.

### RNA-seq data download and expression analysis

RNA-seq datasets corresponding to various stress conditions in Amaranth were retrieved from the NCBI Sequence Read Archive (SRA) submitted under Bio Projects PRJNA263128 and PRJNA65409. These include samples from pre-linear stage (SRR1598909), stem (SRR1598910), flower (SRR1598911), root (SRR1598913), cotyledons (SRR1598915), seed (SRR1598916) and pigmented red stem (SRR172680), drought stress (SRR1598914), salt stress (SRR183483), bacterial infection (SRR172679), waterlogging (SRR172677), insect herbivory (SRR172676). Transcript abundance for NAC genes was quantified using Salmon (v1.9.0)^43^ in quasi-mapping-based mode, with the NAC coding sequences as the reference transcriptome. The quantification outputs were imported and summarised using the tximport package in R, followed by differential gene expression analysis using DESeq2. A variance-stabilising transformation was applied to normalise the expression values. Heatmaps were generated in R using the pheatmap package to visualise the relative expression patterns of 70 NAC genes across five abiotic and biotic stress conditions and seven tissues. This approach enabled the identification of condition-specific upregulation and downregulation of NAC genes involved in stress responses.

### Plant material and drought stress treatment

Seeds of *Amaranthus hypochondriacus* genotype “Annapurna” were obtained from the National Gene Bank at ICAR–NBPGR, New Delhi, under the accession number IC42258-1. This released cultivar was sourced from the national gene bank to ensure authenticity**.** Plants were grown under controlled greenhouse conditions (25 ± 1 °C, 16 h light/8 h dark) at ICAR-NBPGR, New Delhi. After 30 days, uniform seedlings at the four-leaf stage were transferred to pots containing a coco peat: vermiculite mixture (2:1). Drought stress was imposed using the gravimetric method described by Imakumbili et al. (2021)^54^, with water regimes of 100%, 60%, 30%, and 10% field capacity to simulate control, moderate, severe, and water-deficit conditions, respectively. Leaf samples were harvested at 0, 15, and 30 days post-treatment in three biological replicates, immediately frozen in liquid nitrogen, and stored at –80 °C for RNA extraction.

### RNA isolation, cDNA preparation and RT-qPCR analysis

Total RNA was extracted from drought-stressed leaf tissues using the RNeasy® Plant Mini Kit (Qiagen, Germany), following the manufacturer’s protocol. Single-strand cDNA synthesis was performed using 1 µg of RNA with the Verso cDNA Synthesis Kit (Thermo Scientific). Gene-specific primers were designed using Primer3^55^ and validated by agarose gel electrophoresis. *Actin* (Accession No.: KJ634809) was used as the internal reference gene. RT-qPCR was conducted on a BIO-RAD CFX96 system using SYBR Green chemistry under the following conditions: 95 °C for 4 min, followed by 40 cycles of 95 °C for 20 s, 58 °C for 45 s, and 72 °C for 20 s. Each reaction included two biological and three technical replicates. Relative expression levels were calculated using the 2–ΔΔCT method^56^, and fold changes were visualised using ggplot2 in R with error bars representing mean ± SD.

## Supplementary Information

Below is the link to the electronic supplementary material.

Supplementary Material 1

## Data Availability

The dataset of 70 identified AhypNAC gene sequences (genes including promoter region and the encoded protein) is available for download in FASTA format from FigShare at https://figshare.com/s/cf4aedbf2e95b3959690.
