# Supplementary material for: Genome-wide analysis of NAC transcription factors in grain amaranth reveals structural diversity and regulatory features
Source: Sci Rep. 2025 Nov 14;15:39968. doi: 10.1038/s41598-025-23630-7 (PMC12618503; doi:10.1038/s41598-025-23630-7)
Supplement: Supplementary file 1 — Supplementary Material 1 [file 41598_2025_23630_MOESM1_ESM.docx]

**Title: Genome-wide analysis of NAC transcription factors in grain amaranth reveals structural diversity and regulatory features**

**Supplementary Material:**

**Table S1:** List of 70 NAC genes in *A. hypochondriacus* with their chromosome number, start position, and end position in base pairs (bp), named from *AhypNAC01* to *AhypNAC70*.

| **S. No** | **Phytozome ID** | **Renamed ID** | **Start (bp)** | **End (bp)** | **Chr. number** |
| --- | --- | --- | --- | --- | --- |
| 1 | AH000003-RA | *AhypNAC01* | 46709 | 49824 | 1 |
| 2 | AH000261-RA | *AhypNAC02* | 2467494 | 2476219 | 1 |
| 3 | AH000746-RA | *AhypNAC03* | 8659311 | 8661541 | 1 |
| 4 | AH000846-RA | *AhypNAC04* | 10866223 | 10875360 | 1 |
| 5 | AH001077-RA | *AhypNAC05* | 19341870 | 19345900 | 1 |
| 6 | AH001281-RA | *AhypNAC06* | 23670674 | 23678760 | 1 |
| 7 | AH001384-RA | *AhypNAC07* | 25222251 | 25231402 | 1 |
| 8 | AH002239-RA | *AhypNAC08* | 35346029 | 35353197 | 1 |
| 9 | AH002344-RA | *AhypNAC09* | 36603998 | 36606491 | 1 |
| 10 | AH002840-RA | *AhypNAC10* | 3588288 | 3590777 | 2 |
| 11 | AH002860-RA | *AhypNAC11* | 3731959 | 3735340 | 2 |
| 12 | AH003216-RA | *AhypNAC12* | 8211774 | 8213202 | 2 |
| 13 | AH004236-RA | *AhypNAC13* | 31213245 | 31216274 | 2 |
| 14 | AH004356-RA | *AhypNAC14* | 32319628 | 32321520 | 2 |
| 15 | AH004573-RA | *AhypNAC15* | 34386167 | 34388320 | 2 |
| 16 | AH005032-RA | *AhypNAC16* | 3301621 | 3303952 | 3 |
| 17 | AH005066-RA | *AhypNAC17* | 3624333 | 3627241 | 3 |
| 18 | AH005222-RA | *AhypNAC18* | 5508507 | 5510547 | 3 |
| 19 | AH005265-RA | *AhypNAC19* | 6020458 | 6024119 | 3 |
| 20 | AH005423-RA | *AhypNAC20* | 7847109 | 7855749 | 3 |
| 21 | AH006071-RA | *AhypNAC21* | 19980631 | 19981798 | 3 |
| 22 | AH006230-RA | *AhypNAC22* | 26078141 | 26080123 | 3 |
| 23 | AH006231-RA | *AhypNAC23* | 26091511 | 26093517 | 3 |
| 24 | AH006232-RA | *AhypNAC24* | 26106480 | 26113916 | 3 |
| 25 | AH007051-RA | *AhypNAC25* | 6822048 | 6827405 | 4 |
| 26 | AH007342-RA | *AhypNAC26* | 13951954 | 13955248 | 4 |
| 27 | AH007854-RA | *AhypNAC27* | 24083190 | 24087359 | 4 |
| 28 | AH007856-RA | *AhypNAC28* | 24108697 | 24119686 | 4 |
| 29 | AH007909-RA | *AhypNAC29* | 24644665 | 24650360 | 4 |
| 30 | AH008045-RA | *AhypNAC30* | 26006652 | 26007506 | 4 |
| 31 | AH008046-RA | *AhypNAC31* | 26040516 | 26044663 | 4 |
| 32 | AH008092-RA | *AhypNAC32* | 26433749 | 26436738 | 4 |
| 33 | AH008419-RA | *AhypNAC33* | 1118873 | 1123615 | 5 |
| 34 | AH008488-RA | *AhypNAC34* | 1834144 | 1838717 | 5 |
| 35 | AH009185-RA | *AhypNAC35* | 17017989 | 17019829 | 5 |
| 36 | AH009400-RA | *AhypNAC36* | 21612529 | 21616403 | 5 |
| 37 | AH009474-RA | *AhypNAC37* | 22500626 | 22502264 | 5 |
| 38 | AH009495-RA | *AhypNAC38* | 22831638 | 22836304 | 5 |
| 39 | AH010008-RA | *AhypNAC39* | 4914241 | 4916868 | 6 |
| 40 | AH010009-RA | *AhypNAC40* | 4942791 | 4944198 | 6 |
| 41 | AH010270-RA | *AhypNAC41* | 12222731 | 12233578 | 6 |
| 42 | AH010413-RA | *AhypNAC42* | 15048048 | 15051417 | 6 |
| 43 | AH010633-RA | *AhypNAC43* | 17906631 | 17907687 | 6 |
| 44 | AH010717-RA | *AhypNAC44* | 18920739 | 18925869 | 6 |
| 45 | AH010990-RA | *AhypNAC45* | 22200286 | 22204360 | 6 |
| 46 | AH011364-RA | *AhypNAC46* | 4418126 | 4418655 | 7 |
| 47 | AH011875-RA | *AhypNAC47* | 17381185 | 17385953 | 7 |
| 48 | AH012180-RA | *AhypNAC48* | 21584033 | 21586952 | 7 |
| 49 | AH012226-RA | *AhypNAC49* | 22034158 | 22038976 | 7 |
| 50 | AH012584-RA | *AhypNAC50* | 1539359 | 1549041 | 8 |
| 51 | AH013896-RA | *AhypNAC51* | 21785911 | 21791867 | 8 |
| 52 | AH014445-RA | *AhypNAC52* | 13057129 | 13067903 | 9 |
| 53 | AH014651-RA | *AhypNAC53* | 15702571 | 15706535 | 9 |
| 54 | AH015266-RA | *AhypNAC54* | 22419530 | 22425906 | 9 |
| 55 | AH015775-RA | *AhypNAC55* | 12409671 | 12412394 | 10 |
| 56 | AH015793-RA | *AhypNAC56* | 12781245 | 12783865 | 10 |
| 57 | AH016668-RA | *AhypNAC57* | 116085 | 125894 | 11 |
| 58 | AH017051-RA | *AhypNAC58* | 11627980 | 11633524 | 11 |
| 59 | AH017360-RA | *AhypNAC59* | 15514326 | 15516711 | 11 |
| 60 | AH017551-RA | *AhypNAC60* | 17544114 | 17547617 | 11 |
| 61 | AH017564-RA | *AhypNAC61* | 17721202 | 17723191 | 11 |
| 62 | AH017718-RA | *AhypNAC62* | 19363557 | 19374002 | 11 |
| 63 | AH018188-RA | *AhypNAC63* | 2107275 | 2110038 | 12 |
| 64 | AH018336-RA | *AhypNAC64* | 3686564 | 3689048 | 12 |
| 65 | AH018557-RA | *AhypNAC65* | 6189417 | 6196152 | 12 |
| 66 | AH020926-RA | *AhypNAC66* | 11164998 | 11181263 | 14 |
| 67 | AH021139-RA | *AhypNAC67* | 14631549 | 14634819 | 14 |
| 68 | AH021619-RA | *AhypNAC68* | 19568320 | 19573598 | 14 |
| 69 | AH021789-RA | *AhypNAC69* | 959006 | 962605 | 15 |
| 70 | AH023417-RA | *AhypNAC70* | 9486029 | 9489650 | 16 |

**Table S2:** Result summary table of the *AhypNAC* gene duplication on the *A. hypochondriacus* genome.

| **S. No** | **Gene** | **Duplication** | **Chromosome number** |
| --- | --- | --- | --- |
| 1 | *AhypNAC01* | Dispersed | 1 |
| 2 | *AhypNAC02* | Dispersed | 1 |
| 3 | *AhypNAC05* | Dispersed | 1 |
| 4 | *AhypNAC06* | Dispersed | 1 |
| 5 | *AhypNAC07* | Dispersed | 1 |
| 6 | *AhypNAC57* | Dispersed | 1 |
| 7 | *AhypNAC58* | Dispersed | 1 |
| 8 | *AhypNAC59* | Dispersed | 1 |
| 9 | *AhypNAC64* | Dispersed | 1 |
| 10 | *AhypNAC20* | Dispersed | 2 |
| 11 | *AhypNAC21* | Dispersed | 2 |
| 12 | *AhypNAC25* | Dispersed | 2 |
| 13 | *AhypNAC26* | Dispersed | 2 |
| 14 | *AhypNAC67* | Dispersed | 2 |
| 15 | *AhypNAC70* | Dispersed | 2 |
| 16 | *AhypNAC29* | Dispersed | 3 |
| 17 | *AhypNAC33* | Dispersed | 3 |
| 18 | *AhypNAC34* | Dispersed | 3 |
| 19 | *AhypNAC35* | Dispersed | 3 |
| 20 | *AhypNAC36* | Dispersed | 3 |
| 21 | *AhypNAC37* | Dispersed | 3 |
| 22 | *AhypNAC41* | Dispersed | 3 |
| 23 | *AhypNAC42* | Dispersed | 3 |
| 24 | *AhypNAC43* | Dispersed | 3 |
| 25 | *AhypNAC45* | Dispersed | 4 |
| 26 | *AhypNAC46* | Dispersed | 4 |
| 27 | *AhypNAC47* | Dispersed | 4 |
| 28 | *AhypNAC48* | Dispersed | 4 |
| 29 | *AhypNAC49* | Dispersed | 4 |
| 30 | *AhypNAC51* | Dispersed | 4 |
| 31 | *AhypNAC53* | Dispersed | 4 |
| 32 | *AhypNAC54* | Dispersed | 4 |
| 33 | *AhypNAC03* | WGD or Segmental | 5 |
| 34 | *AhypNAC04* | WGD or Segmental | 5 |
| 35 | *AhypNAC08* | WGD or Segmental | 5 |
| 36 | *AhypNAC09* | WGD or Segmental | 5 |
| 37 | *AhypNAC55* | WGD or Segmental | 5 |
| 38 | *AhypNAC56* | WGD or Segmental | 5 |
| 39 | *AhypNAC60* | WGD or Segmental | 6 |
| 40 | *AhypNAC61* | WGD or Segmental | 6 |
| 41 | *AhypNAC62* | WGD or Segmental | 6 |
| 42 | *AhypNAC63* | WGD or Segmental | 6 |
| 43 | *AhypNAC65* | WGD or Segmental | 6 |
| 44 | *AhypNAC66* | WGD or Segmental | 6 |
| 45 | *AhypNAC68* | WGD or Segmental | 6 |
| 46 | *AhypNAC10* | WGD or Segmental | 7 |
| 47 | *AhypNAC11* | WGD or Segmental | 7 |
| 48 | *AhypNAC12* | WGD or Segmental | 7 |
| 49 | *AhypNAC69* | WGD or Segmental | 7 |
| 50 | *AhypNAC13* | WGD or Segmental | 8 |
| 51 | *AhypNAC14* | WGD or Segmental | 8 |
| 52 | *AhypNAC15* | WGD or Segmental | 9 |
| 53 | *AhypNAC16* | WGD or Segmental | 9 |
| 54 | *AhypNAC17* | WGD or Segmental | 9 |
| 55 | *AhypNAC18* | WGD or Segmental | 10 |
| 56 | *AhypNAC19* | WGD or Segmental | 10 |
| 57 | *AhypNAC27* | WGD or Segmental | 11 |
| 58 | *AhypNAC32* | WGD or Segmental | 11 |
| 59 | *AhypNAC38* | WGD or Segmental | 11 |
| 60 | *AhypNAC39* | WGD or Segmental | 11 |
| 61 | *AhypNAC40* | WGD or Segmental | 11 |
| 62 | *AhypNAC44* | WGD or Segmental | 11 |
| 63 | *AhypNAC50* | WGD or Segmental | 12 |
| 64 | *AhypNAC52* | WGD or Segmental | 12 |
| 65 | *AhypNAC22* | Tandem | 12 |
| 66 | *AhypNAC23* | Tandem | 14 |
| 67 | *AhypNAC24* | Tandem | 14 |
| 68 | *AhypNAC30* | Tandem | 14 |
| 69 | *AhypNAC31* | Tandem | 15 |
| 70 | *AhypNAC28* | Proximal | 16 |

**Table S3.** Analysis of non-synonymous mutations (Ka), synonymous mutations (Ks), and the calculated evolutionary pressure (Ka/Ks ratio) among 17 segmentally duplicated *AhypNAC* gene pairs.

| Seq_1 | Seq_2 | Ka | Ks | Ka/Ks |
| --- | --- | --- | --- | --- |
| *AhypNAC03* | *AhypNAC09* | 0.197 | 1.548 | 0.127 |
| *AhypNAC04* | *AhypNAC08* | 0.138 | 0.724 | 0.190 |
| *AhypNAC56* | *AhypNAC61* | 0.311 | 1.419 | 0.219 |
| *AhypNAC55* | *AhypNAC17* | 0.231 | 1.904 | 0.121 |
| *AhypNAC60* | *AhypNAC11* | 0.174 | 0.585 | 0.298 |
| *AhypNAC61* | *AhypNAC10* | 0.148 | 0.506 | 0.293 |
| *AhypNAC61* | *AhypNAC16* | 0.447 | 2.067 | 0.216 |
| *AhypNAC65* | *AhypNAC12* | 0.070 | 0.236 | 0.298 |
| *AhypNAC63* | *AhypNAC38* | 0.339 | 1.794 | 0.189 |
| *AhypNAC68* | *AhypNAC39* | 0.329 | 1.878 | 0.175 |
| *AhypNAC66* | *AhypNAC52* | 0.124 | 0.447 | 0.278 |
| *AhypNAC69* | *AhypNAC50* | 0.228 | 0.773 | 0.295 |
| *AhypNAC14* | *AhypNAC16* | 0.231 | 0.538 | 0.430 |
| *AhypNAC15* | *AhypNAC18* | 0.081 | 0.608 | 0.133 |
| *AhypNAC13* | *AhypNAC19* | 0.086 | 0.601 | 0.143 |
| *AhypNAC27* | *AhypNAC44* | 0.256 | 1.733 | 0.148 |
| *AhypNAC32* | *AhypNAC40* | 0.108 | 0.559 | 0.193 |

**Table S4.** TMHMM-based prediction of transmembrane helix regions in AhypNAC proteins.

| **Gene ID** | **Renamed ID** | **Length (aa)** | **Outside TM** | **TM helix** | **Inside TM** |
| --- | --- | --- | --- | --- | --- |
| AH000003-RA | *AhypNAC01* | 406 | 406-406 | 386-405 | 1-385 |
| AH007051-RA | *AhypNAC25* | 545 | 1-520 | 521-543 | 544-545 |
| AH010990-RA | *AhypNAC45* | 531 | 1-506 | 507-529 | 530-531 |
| AH011875-RA | *AhypNAC47* | 606 | 1-575 | 576-598 | 599-606 |
| AH013896-RA | *AhypNAC51* | 368 | 1-344 | 345-367 | 368-368 |
| AH021139-RA | *AhypNAC67* | 634 | 1-601 | 602-624 | 625-634 |

**Table S5:** Predicted targets of Ahyp-miR164a among NAC genes in *A. hypochondriacus*

| **miRNA** | **Target** | **Expectation** | **UPE** | **Cleavage Site** | **Inhibition** |
| --- | --- | --- | --- | --- | --- |
| Ahyp-miR164a | *AhypNAC02* | 0.5 | 7.497 | 683–703 | Cleavage |
| Ahyp-miR164a | *AhypNAC10* | 0.5 | 7.512 | 569–589 | Cleavage |
| Ahyp-miR164a | *AhypNAC61* | 1.5 | 14.782 | 617–637 | Cleavage |
| Ahyp-miR164a | *AhypNAC56* | 3 | 17.821 | 629–649 | Cleavage |

**Table S6.** Differentially expressed NAC genes under various abiotic and biotic stress conditions.

| **Stress Condition** | **Upregulated Genes** | **Downregulated Genes** |
| --- | --- | --- |
| Drought Stress | *AhypNAC23, AhypNAC24, AhypNAC32* | *AhypNAC03, AhypNAC70* |
| Salt Stress | *AhypNAC06, AhypNAC17, AhypNAC51* | *AhypNAC13, AhypNAC19, AhypNAC38, AhypNAC63, AhypNAC66, AhypNAC69* |
| Insect Herbivory | *AhypNAC17, AhypNAC65* | *NIL* |
| Bacterial Infection | *AhypNAC17, AhypNAC65, AhypNAC69* | *AhypNAC15, AhypNAC25* |
| Waterlogging | *AhypNAC17, AhypNAC69* | *NIL* |

**Table S7.**  List of primers used for qRT-PCR validation under drought condition.

| **S. No.** | **Gene name** | **Primer sequence** | **Length** | **Tm** | **GC%** | **Product Size (bp)** |
| --- | --- | --- | --- | --- | --- | --- |
| 1 | *AhypNAC23_F* | GCATTGGAGAGCTGTGGTC | 19 | 58.52 | 57.89 | 242 |
|  | *AhypNAC23_R* | TGGGCTCCAAAATTAGGGCT | 20 | 59.29 | 50 |  |
| 2 | *AhypNAC24_F* | AGTGGGTTGGAAGAAATGCT | 20 | 57.32 | 45 | 171 |
|  | *AhypNAC24_R* | CGACAAACCACCCACTTCTT | 20 | 58.32 | 50 |  |
| 3 | *AhypNAC03_F* | ACTTTGAGTTACCTGGGTTTAGA | 23 | 57.1 | 39.13 | 150 |
|  | *AhypNAC03_R* | TTCTCTTTCTCCTATTTTCGCCA | 23 | 57.84 | 39.13 |  |
| 4 | *AhypNAC70_F* | ATGACCCTTGGGATCTTGCA | 20 | 59 | 50 | 236 |
|  | *AhypNAC70_R* | TCCAGCACTTCCACGGTAAT | 20 | 59.02 | 50 |  |
| 5 | *Actin_F* | CGTGACCTGACTGATTACCTTA | 22 | 79.2 | 45.5 | 178 |
|  | *Actin_R* | GCTCGTAGTTCTTCTCAATGGC | 22 | 79.4 | 50 |  |


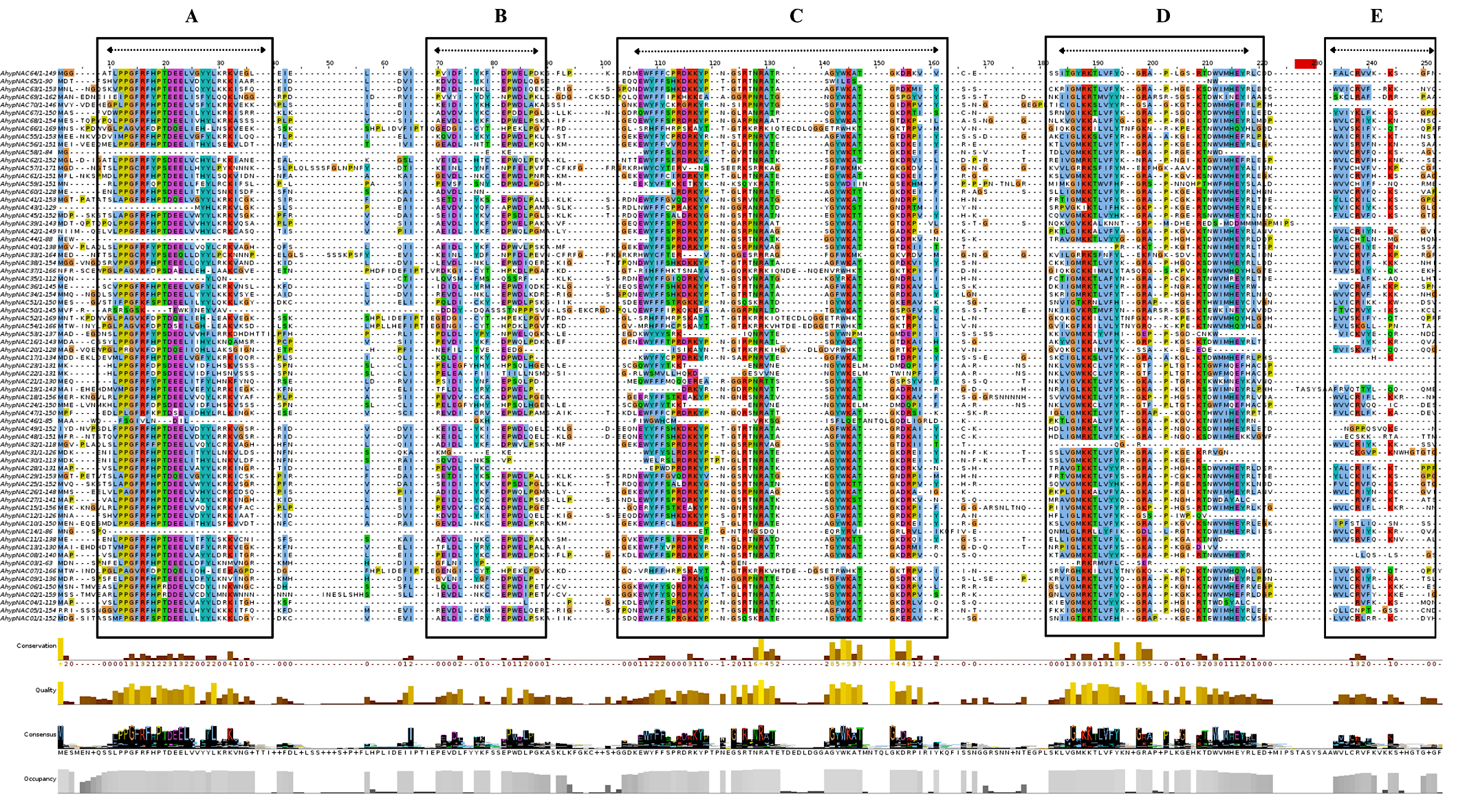


**Figure S1:** Sequence alignment of all 70 identified NAC genes of *A. hypochondriacus* highlights subdomains A to E, represented by five black boxes, each corresponding to a subdomain. Amino acid conservation, quality, and consensus are illustrated using a bar graph, where the height of each bar indicates the level of conservation, ranging from lower to higher.


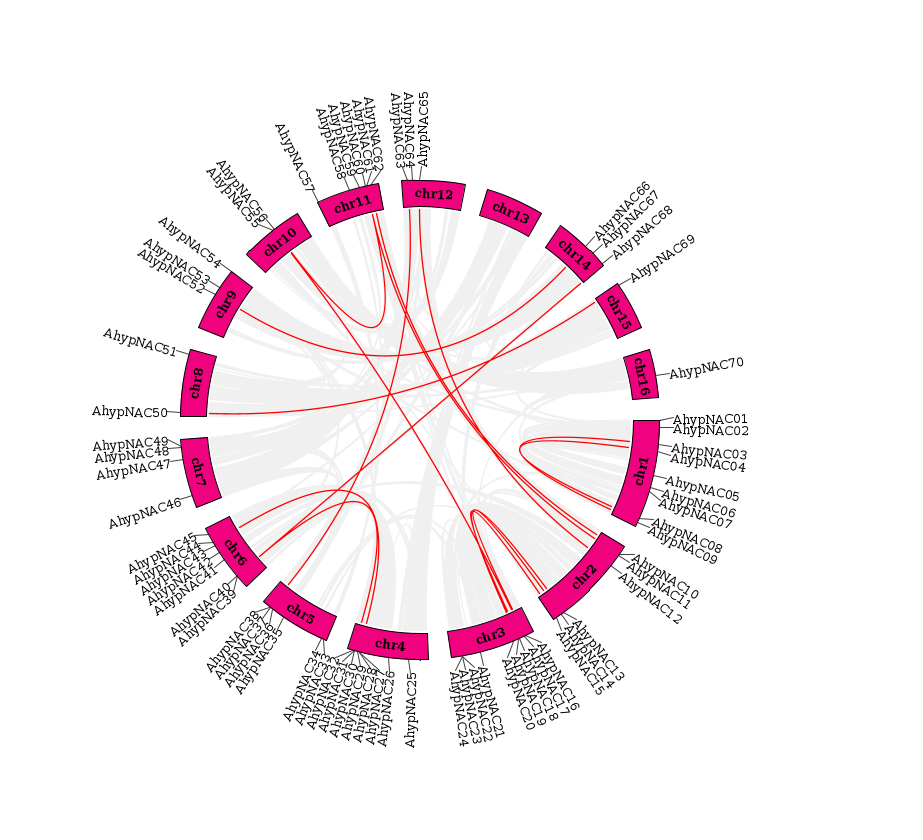


**Figure S2:** Circos plot showing the inter-chromosomal relationships of NAC genes within its genome. Grey lines depict all synteny blocks within the Amaranth genome, while red lines highlight segmental duplicated NAC gene pairs.

| **Motif**  **Number** | **Motifs Sequence** |
| --- | --- |
| 1 | RKYPNGSRTNRATEAGYWKATGKDRPI |
| 2 | GFRFHPTDEELVVYYLKRKVNGRKIDLDIIPEVD |
| 3 | APVGMKKTWVMYEGR |
| 4 | LYKFEPWDLPGKAKJGEKEWY |
| 5 | KLDDWVLCRVFKKSGSGKKPDESAGSSSDE |
| 6 | TIEGEDGICYTHPEKLPGVTKDGLSRHFFHRPSKAYTTGTRKRRKIQTDC |
| 7 | TRWHKTGKTRPVLVNGIQKGCKKILVLYT |
| 8 | SSHDHHHHHNHDHHZ |
| 9 | NNNNNNNNNNNNNNNN |
| 10 | LGQDEEEKEGELVVSKIFYQTQPRQCGW |

**Figure S3:** Top 10 conserved motifs with lengths ranging from 15 to 50 amino acids identified in the AhypNAC Gene Using MEME Suite. Each position is represented by a stack of letters, with stack height indicating the information content in bits (reflecting conservation and specificity), and individual letter height signifying the nucleotide's probability at that position, scaled by the stack's total information content.
